# What would Gregory Maskarinec and Paul Farmer do?

**DOI:** 10.1002/puh2.189

**Published:** 2024-10-07

**Authors:** Seiji Yamada

**Affiliations:** ^1^ Department of Family Medicine and Community Health University of Hawaii Honolulu Hawaii USA

In this letter, I propose that two of our leading lights in public health would have opposed the genocide in Gaza—if they were with us today. The last two iterations of the Humanism, Empathy, Social Justice, and Global Health Symposia at the University of Hawaii (UH) John A. Burns School of Medicine (JABSOM) were coordinated by Gregory Maskarinec, cultural anthropologist and Director of the Office of Global Health and International Medicine at the time. Paul Farmer joined us remotely from Rwanda for the February 2022 Symposium, delivering two talks in 2 days. Paul died 4 days after his second talk—an incalculable loss for the world. Gregory had been ill since 2020 and was not expected to live much longer after the February 2022 Symposium. Paul had expressed his sadness at Gregory's impending demise, but ironically, he died ahead of Gregory, who passed away in June 2022. Another incalculable loss.

For this essay, I have drawn on the conversations, correspondence, their writings, and an article that I wrote together with Gregory and Paul. I am led to believe that at the current juncture, Gregory and Paul would have spoken out against the genocidal assault on the Palestinian people.

## PAUL FARMER'S EARLY WORK

Paul Farmer's early work focused on the people of Haiti and their health and illness. He advocated for historically deep and geographically broad inquiry, examining the role of large‐scale social forces in shaping health and illness. Caring for patients during the brutal Jean‐Claude “Baby Doc” Duvalier dictatorship, he was attuned to violations of human rights. He wrote about one of his patients who was brutally beaten to death by the Tonton Macoutes, Duvalier's death squad.

In *Pathologies of Power* (2003), Farmer began to examine human rights abuses outside of Haiti, writing about the exhumation of the victims of genocide of the indigenous people of Guatemala by a series of military dictatorships [1]. Some 200,000 Mayan people were killed, with the highest rate of killings perpetrated under the rule of General Efraín Ríos Montt (1982–1983). Israel provided much of the armaments and training [2].

## THE 1994 RWANDA GENOCIDE

The 1994 Rwanda genocide left 500–80,000 dead and the country devastated. Philip Gourevitch's book *We wish to inform you that tomorrow we will be killed with our families* was published in 1998 [3]. I read it that fall as I participated in a graduate seminar in anthropology taught by Gregory Maskarinec. “Social Suffering” was a class that examined ways that suffering, pain, disease, and their consequences were expressed and addressed differently in different cultures.

In 2005, Farmer delivered the Tanner Lecture in Human Values on “Never again? Reflections on human values and human rights” [4]. He also began working with the Rwandan government in 2005. When he visited us in person in 2007, delivering lectures at the Hawaii Academy of Family Physicians, the JABSOM, and the UH at Mānoa, he told us about his work in Rwanda. He moved his family and his base of operations to Rwanda, also starting the University of Global Health Equity there in 2005.

## THE 2003 ASSAULT ON IRAQ

The March 2003 Anglo‐American assault on and subsequent occupation of Iraq imposed an excess burden of morbidity and mortality on the people of Iraq. In “Casualties: narrative and images of the war on Iraq,” Mary Kay Smith Fawzi, Gregory Maskarinec, Paul Farmer, and I asserted that public health and medical practitioners have a responsibility to seek out accounts and images of the suffering of the victims of the assault [5]. We explored possible responses to narrative, and images of this suffering and outlined the sorts of responses engendered by three perspectives—charity, development, and social justice. We asserted that the suffering of the people of Iraq should spur a social justice response from the health community to alleviate the situation and prevent unnecessary suffering.

## CONCLUSIONS

I believe that those who have passed are still with us, as long as we keep them alive in our memories. I thus seek to keep Gregory and Paul with us by having them weigh in on our present circumstances. After Paul's passing, Gregory and I wrote about “What Paul Farmer book should you read?” Regarding Chapter 5 of *Pathologies of power*, we wrote

On what basis, then, should care to the poor be delivered: *through charity, through development, or through social justice*? Drawing upon liberation theology, Farmer favors social justice, that view that the poor are not poor because of individual shortcomings but because they are victims of structural violence, the large‐scale social forces that create and enforce their poverty. In summary, Farmer challenges us to rethink what has now become the fundamental basis of the practice of medicine, that the best care is reserved for those who can afford it. Would it that there were a hundred Paul Farmers to take first‐class medicine to the poor of the world. Since there was only one, however, it is now up to the rest of us [6].

I think that Paul would let us substitute “survivors of militarized, genocidal assault” for “the poor.” The article was Gregory and my last collaboration in the material world, but that does not mean that we don't continue to work together.

## AUTHOR CONTRIBUTIONS

*Conceptualization; writing—original draft; writing—review and editing*: Seiji Yamada.

## CONFLICT OF INTEREST STATEMENT

The author declares no conflicts of interest.

## FUNDING INFORMATION

No funding supported this work.
